# Doubling the Carbonate-Binding Capacity of Nanojars by the Formation of Expanded Nanojars

**DOI:** 10.3390/molecules26113083

**Published:** 2021-05-21

**Authors:** Wisam A. Al Isawi, Gellert Mezei

**Affiliations:** Department of Chemistry, Western Michigan University, Kalamazoo, MI 49008, USA; wisam.alisawi@wmich.edu

**Keywords:** supramolecular chemistry, inverse coordination, anion binding, nanojar, copper–pyrazolate complex, carbonate binding

## Abstract

Anion binding and extraction from solutions is currently a dynamic research topic in the field of supramolecular chemistry. A particularly challenging task is the extraction of anions with large hydration energies, such as the carbonate ion. Carbonate-binding complexes are also receiving increased interest due to their relevance to atmospheric CO_2_ fixation. Nanojars are a class of self-assembled, supramolecular coordination complexes that have been shown to bind highly hydrophilic anions and to extract even the most hydrophilic ones, including carbonate, from water into aliphatic solvents. Here we present an expanded nanojar that is able to bind two carbonate ions, thus doubling the previously reported carbonate-binding capacity of nanojars. The new nanojar is characterized by detailed single-crystal X-ray crystallographic studies in the solid state and electrospray ionization mass spectrometric (including tandem MS/MS) studies in solution.

## 1. Introduction

**Figure 1:**
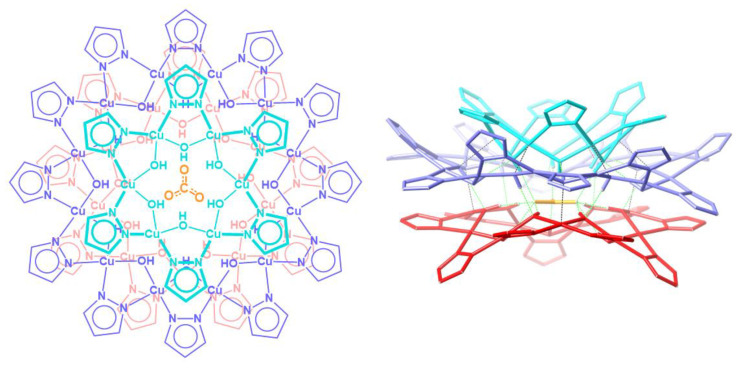
Schematic representation (**left**, **top-view**) of the crystal structure (**right**, **side-view**) of the [CO_3_⊂{Cu^II^(*μ*-OH)(*μ*-pz)}_6 + 12 + 9_]^2−^ nanojar [1].

Nanojars are a family of supramolecular coordination complexes that form from a solution of Cu^2+^, OH^−^ and pyrazolate (pz = C_3_H_3_N_2_^−^) ions in the presence of a hydrophilic anion, such as carbonate [1], sulfate [2], phosphate [3], arsenate [3] or chloride [4]. The anion templates the formation of {*cis*-Cu^II^(*μ*-OH)(*μ*-pz)}_x_ metallamacrocycles (*x* = 6–14, except 11). Three (in the case of carbonate, sulfate, phosphate, arsenate) or four (in the case of chloride) of these metallamacrocycles self-assemble around a central anion into nanojars of the formula [anion⊂{Cu^II^(*μ*-OH)(*μ*-pz)}_n_] (*n* = 27–33), via inter-metallamacrocycle and anion-metallamacrocycle hydrogen bonding, as well as inter-metallamacrocycle Cu∙∙∙O interactions. The incarcerated anion appears to be crucial for the formation of nanojars, as the neutral nanojar host does not exist on its own without an anion guest. Figure 1 illustrates the structure of the nanojar with *n* = 27.

The recognition and binding of anions has been receiving increased interest in recent years [5,6,7], as the supramolecular binding of anions finds applications in anion sensing, extraction and separation of anions, transmembrane anion transport and anion-driven architectonics and organocatalysis [8]. We have recently shown that nanojars bind the incarcerated oxoanions (carbonate, sulfate, phosphate, arsenate) with unprecedented strength by wrapping a multitude of hydrogen bonds around the anion and totally isolating it from its surrounding medium (as in the sulfate [9] and phosphate [10] binding proteins). Indeed, an aqueous Ba^2+^ solution is unable to precipitate the corresponding barium salt (e.g., BaSO_4_, *K*_sp_ = 1.08 × 10^−10^ at 25 °C in H_2_O) when stirred with a solution of the nanojars. We have also demonstrated that nanojars are able to transfer these anions, including one of the most hydrophilic ones, carbonate, from water into aliphatic solvents [11]. Thus, nanojars can be used as extraction agents for the removal of such anions from contaminated aqueous media by liquid–liquid extraction [12].

Herein we report the serendipitous discovery that upon addition of 1,10-phenathroline into the nanojar-forming reaction mixture, expanded nanojars form that bind two carbonate ions instead of one, thus doubling the carbonate-binding capacity of nanojars. As described below, the binding of the second carbonate ion by four copper-centers (*μ*_4_-CO_3_) provides for an interesting new example of an inverse coordination complex, wherein the bridging ligand is the coordination center surrounded by metal ions [13,14,15,16,17,18,19,20].

## 2. Results and Discussion

Nanojars have so far been characterized almost exclusively with tetrabutylammonium as counterion, such as in (Bu_4_N^+^)_2_[CO_3_^2−^⊂{Cu^II^(*μ*-OH)(*μ*-pz)}_6+12+9_] (**1**), with the single exception of (K^+^ ⊂ 18-crown-6)_2_[SO_4_^2−^⊂{Cu^II^(*μ*-OH)(*μ*-pz)}_8+14+9_] [2]. In an attempt to prepare nanojars with [Cu(phen)_3_]^2+^ formed in situ as the countercation, a mixture of CuSO_4_·5H_2_O, pyrazole, NaOH and 1,10-phenanthroline (phen) was stirred in tetrahydrofuran (THF). After filtration and evaporation, the resulting product was crystallized from a nitrobenzene solution by heptane vapor diffusion. Instead of the expected [Cu(phen)_3_][SO_4_⊂{Cu^II^(*μ*-OH)(*μ*-pz)}_n_], X-ray crystallography showed the formation of neutral, expanded nanojars, [CO_3_⊂{Cu_29_(*μ*-OH)_27_(*μ*-pz)_27_(phen)_2_(*μ*_4_-CO_3_)(H_2_O)}] (**2**). Atmospheric CO_2_ was apparently absorbed during the reaction, leading to the binding of two CO_3_^2−^ ions by the resulting nanojar. After rationalizing the obtained structure, the reaction was also repeated using Cu(NO_3_)_2_·2.5H_2_O and Na_2_CO_3_·H_2_O instead of CuSO_4_·5H_2_O.

### 2.1. Crystallographic Description

**Figure 2:**
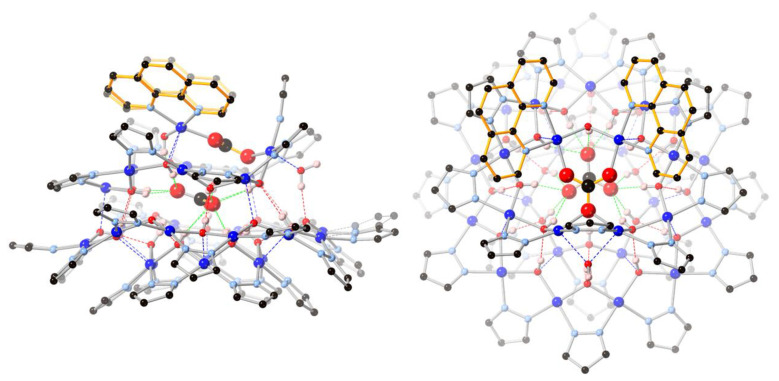
Ball-and-stick representation of the crystal structure of **2**(C_6_H_5_NO_2_)_6.74_(C_7_H_16_)_0.76_: (**left**) side-view; (**right**) top-view. Color code: dark blue—Cu; light blue—N; red—O; black—C; pink—H. The 1,10-phenanthroline moieties are highlighted in orange. The nitrobenzene/heptane solvent molecules and C–H hydrogen atoms are omitted for clarity, and only the major components of the disordered carbonate and pyrazolate moieties are shown.

Located on a general position, nanojar **2** (triclinic, Pī) has pseudo-mirror symmetry (Figure 2). Its structure is closely related to that of **1**, in which three neutral [*cis*-Cu^II^(*μ*-OH)(*μ*-pz)]_n_ rings, with a larger one (*n* = 12) sandwiched by two smaller ones (*n* = 6 and 9), define the nanojar, with its cavity occupied by an incarcerated carbonate ion (Figure 1). The same Cu_6_ + Cu_12_ + Cu_9_ ring combination is found in both **1** and **2**, with the exception that one OH^−^ group of the Cu_9_-ring in **2** is replaced by an O-atom of a second CO_3_^2−^ ion. The central, larger ring is approximately flat, with the pyrazolate units symmetrically alternating slightly above and below the ring mean-plane and not forming hydrogen bonds to the carbonate ion. The smaller side-rings are bowl-shaped, with their pyrazolate moieties pointing away from the central ring and their OH groups pointing toward the center of the nanojar and forming multiple hydrogen bonds with the incarcerated CO_3_^2−^ ion. Although there is no direct bonding between the two smaller rings, they are both involved in multiple H-bonds and weak axial Cu–O interactions with the larger central ring. In the [*cis*-Cu^II^(*μ*-OH)(*μ*-pz)]_n_ rings, Cu–O and Cu–N bond-lengths are within normal ranges, 1.893(3)–2.007(3) and 1.943(6)–2.06(2) Å, respectively (Table 1). While in **1** the 2—charge of the incarcerated carbonate ion is balanced by two Bu_4_N^+^ counterions, in **2** it is the additional bonded [Cu_2_(phen)_2_CO_3_]^2+^ moiety that renders the assembly neutral.

**Figure 3:**
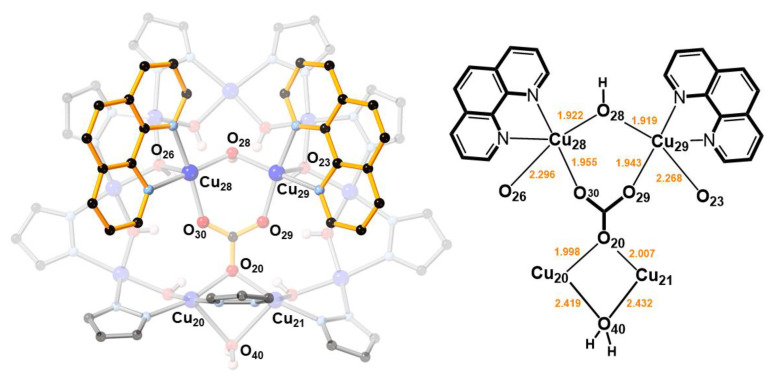
A [Cu_2_(phen)_2_CO_3_]^2+^ moiety binds to the [Cu(*μ*-OH)(*μ*-pz)]_9_ ring in **2** by replacing a OH^−^ group (O28) with an O-atom of its CO_3_^2−^ ion (O20), and by two axial Cu–O bonds (Cu28–O26 and Cu29–O23).

In **2**, two O-atoms of the additional carbonate ion are bound to two Cu^II^(1,10-phen) units, which are bridged by a OH^−^ group (O28) and form weak Cu–O bonds (2.296(3) and 2.268(3) Å) with the Cu_9_-ring (Figure 3). The OH^−^ group (O28) is H-bonded to the central carbonate ion. As a consequence of binding the second carbonate ion, a pyrazolate group of the Cu_9_-ring is pulled away from the Cu_12_-ring, opening up a cavity that becomes occupied by a water molecule. This H_2_O molecule bridges two Cu-atoms of the Cu_9_-ring (Cu···O: 2.419(3) and 2.432(3) Å) and donates a H-bond to a OH-group of the Cu_12_-ring (O40···O8: 2.684(5) Å).

**Figure 4:**
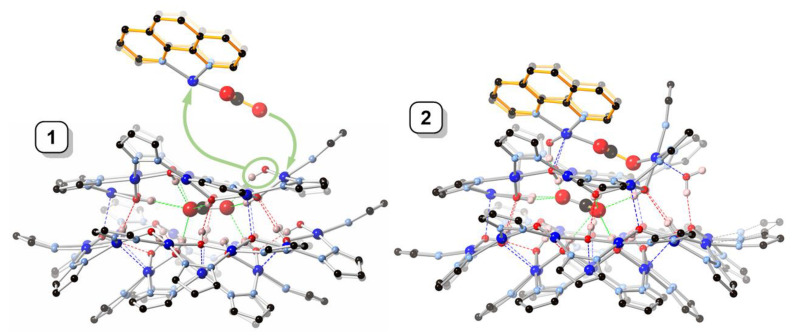
Comparison of the crystal structures of **1** and **2**, illustrating the formation of **2** from **1** by incorporation of a [Cu_2_(phen)_2_CO_3_]^2+^ moiety. Color code: dark blue—Cu; light blue—N; red—O; black—C; pink—H. The 1,10-phenanthroline moieties are highlighted in orange.

While in **1** the central carbonate ion is approximately parallel to the [Cu(*μ*-OH)(*μ*-pz)]_n_ rings, in **2** it is found tilted: the angle between the CO_3_^2−^ and Cu_12_-ring mean-planes is 2.2(1)° in **1** and 22.2(2)° in **2** (Figure 4). As a consequence of the tilting, some of the H-bonding distances to CO_3_^2−^ in **2** (Table 2) are shorter (down to 2.657(5) Å) and others are longer (up to 3.088(5) Å) than in **1** (2.746(5)–2.915(5) Å). Nonetheless, the average of the twelve H-bonds to carbonate (four to each O-atom) is virtually identical in **1** (2.842(5) Å) and **2** (2.838(5) Å). The second CO_3_^2−^ ion in **2** is coordinate-covalently bound to the Cu_9_ ring and the two additional Cu-atoms, almost parallel to the central CO_3_^2−^ ion (angle between mean-planes: 6.6(2)°), with a C···C separation of only 3.071(6) Å. Another very closely-spaced, head-to-head pair of CO_3_^2−^ ions (C···C: 3.664(1), O···O: 1.946(1) Å) has been reported in which both CO_3_^2−^ ions are bound to multiple metal centers [21]. The tetranuclear Cu_4_(*μ*_4_-CO_3_) moiety has also been reported with a few other ligand systems [22,23,24,25,26,27,28,29].

As in **1**, the OH-groups of the Cu_12_-ring in **2** donate twelve alternating H-bonds, six to the Cu_6_-ring (O···O: 2.716(4)–2.786(4) Å, average: 2.742(5) Å) and six to the Cu_9_-ring (O···O: 2.721(4)–2.914(5) Å, average: 2.780(5) Å), with an overall average of 2.762(4) Å. The corresponding overall average of the twelve O···O distances in **1** is virtually identical (2.761(5) Å).

The Cu_6_- and Cu_9_-rings in **2** each form six Cu···O contacts <3.00(8) Å with O-atoms of the Cu_12_-ring (Cu_6_-ring, Cu···O: 2.410(3)–2.609(3) Å, average: 2.476(3) Å; Cu_9_-ring, Cu···O: 2.318(3)–3.079(3) Å; average: 2.698(3) Å). Two Cu-atoms of the Cu_9_-ring (bridged by an O-atom of the second carbonate ion) bind the bridging H_2_O molecule. All other Cu-atoms, including those of the Cu_12_-ring, are at distances larger than 3.196(3) Å from the closest nonbonding O-atoms. Overall, there are twelve Cu···O distances <3.00(8) Å between Cu_n_-rings, with an average of 2.587(3) Å. The corresponding value for **1** is 2.564(4) Å.

**Figure 5:**
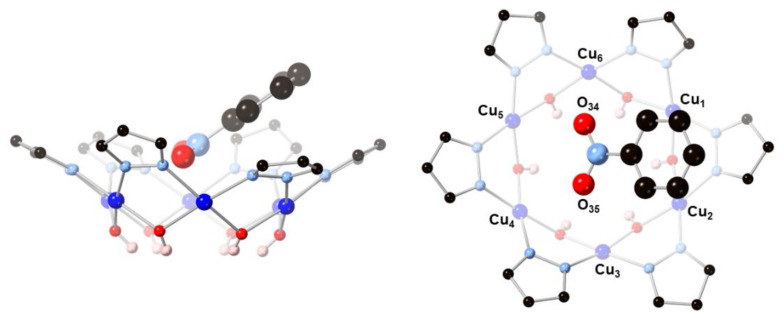
Illustration of the binding of nitrobenzene by the [Cu(*μ*-OH)(*μ*-pz)]_6_ ring of **2**: (**left**) side-view; (**right**) top-view.

In addition to the two carbonate ions, nanojar **2** also binds a nitrobenzene solvent molecule in the outer cavity of the Cu_6_-ring (Figure 5) by a close π-π stacking interaction between the phenyl group and a pyrazolate moiety (centroid···centroid: 3.593(3) Å, angle between mean-planes: 6.7(2)°) and by weak, bifurcated interactions between the O-atoms of the nitro group and four Cu-atoms (Cu···O: 2.647(4) and 3.033(4) Å, and 2.827(4) and 2.844(4) Å, respectively).

**Figure 6:**
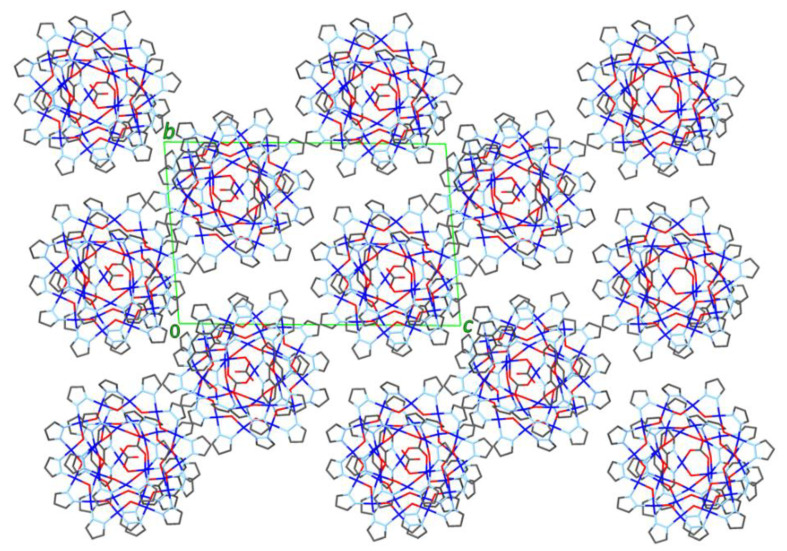
View of the packing diagram of **2** down the *a*-axis (solvent molecules and H-atoms are not shown).

**Figure 7:**
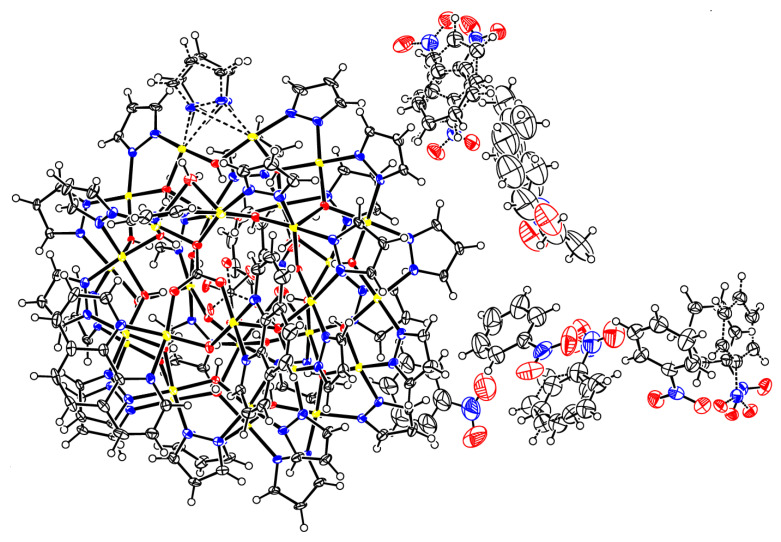
Thermal ellipsoid plot of the crystal structure of **2**(C_6_H_5_NO_2_)_6.74_(C_7_H_16_)_0.76_.

As shown in Figure 6, the close-packing of nanojars leaves relatively large void spaces in the crystal lattice, which are filled by multiple solvent molecules (see also Figure 7). In addition to the nitrobenzene molecule bound in the outer cavity of the Cu_6_-ring of the nanojar, there are five more nitrobenzene molecules filling up the void spaces, as well as a seventh nitrobenzene molecule disordered with a heptane molecule. The presence of aromatic moieties in the included solvent molecules appears to be crucial for the formation of nanojar crystals, as they form multiple aromatic interactions with the nanojar molecules and with each other. Nevertheless, the crystal lattice is not robust: the crystals quickly become opaque and disintegrate if removed from the mother liquor at ambient conditions, requiring low-temperature conditions for X-ray diffraction measurement.

### 2.2. Mass Spectrometric Studies

**Figure 8:**
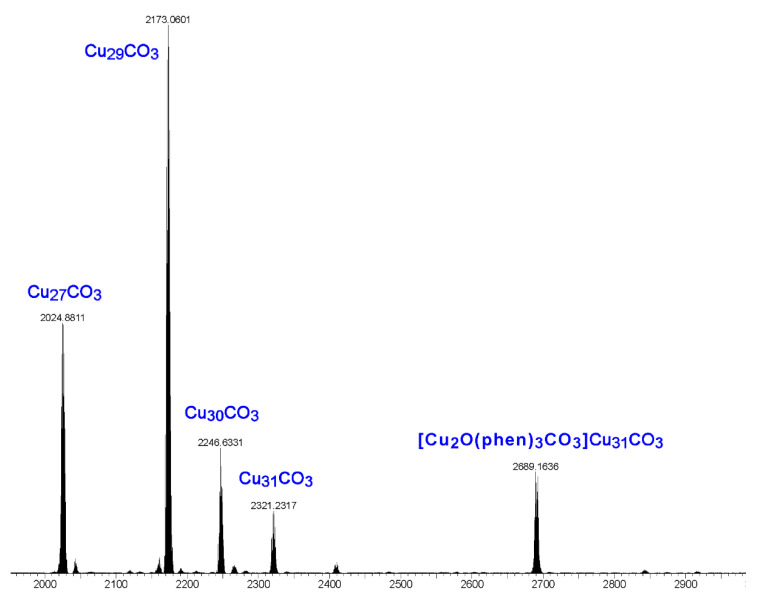
ESI-MS(–) spectrum in DMF of the product obtained from the reaction of copper nitrate, pyrazole, sodium hydroxide, sodium carbonate and 1,10-phenanthroline.

Electrospray ionization mass spectrometry (ESI-MS) studies show that the product obtained from the reaction of copper nitrate, pyrazole, sodium hydroxide, sodium carbonate and 1,10-phenanthroline is a mixture of nanojars (Figure 8). The spectrum shows the following peaks in the negative mode (no nanojar peaks are observable in the positive mode): [CO_3_⊂{Cu(*μ*-OH)(*μ*-pz)}_n_]^2−^ (Cu_n_CO_3_; *n* = 27, *m/z* = 2023; *n* = 29, *m/z* = 2171; *n* = 30, *m/z* = 2245; *n* = 31, *m/z* = 2318) and [{Cu_2_O(phen)_3_CO_3_}CO_3_⊂{Cu(*μ*-OH)(*μ*-pz)}_31_]^2−^ (*m/z* = 2690). The first four peaks correspond to nanojars without the additional [Cu_2_(phen)_2_CO_3_]^2+^ moiety. Obviously, neutral [Cu_2_(phen)_2_(CO_3_)_2_{Cu(OH)(pz)}_n_] nanojar peaks cannot be expected in the mass spectrum. It is apparent that during ionization in the mass spectrometer, the neutral nanojars lose the [Cu_2_(phen)_2_CO_3_]^2+^ moiety and become [CO_3_⊂{Cu(*μ*-OH)(*μ*-pz)}_n_]^2−^. Although the parent, neutral nanojars cannot be observed directly by ESI-MS, several independent observations indirectly support their assumed structure. First, the crystal structure of [Cu_2_(phen)_2_(CO_3_)_2_{Cu(OH)(pz)}_n_] with *n* = 27 has been unambiguously established. In this Cu_6_+Cu_12_+Cu_9_ nanojar, the Cu_2_(phen)_2_CO_3_ moiety is bound to the Cu_9_-ring. Other nanojar sizes, such as with *n* = 29 (Cu_7_+Cu_13_+Cu_9_), *n* = 30 (Cu_8_+Cu_13_+Cu_9_) and *n* = 31 (Cu_8_+Cu_14_+Cu_9_) also have Cu_9_-rings to which the Cu_2_(phen)_2_CO_3_ moiety can bind. Second, a peak corresponding to [Cu^I^(phen)_2_]^+^ (*m/z* = 424) is observed in the positive mode ESI-MS spectrum of the as-synthesized neutral nanojar mixture, which originates from the [Cu_2_(phen)_2_CO_3_]^2+^ moiety detached from the nanojar upon ionization. Third, the neutral nanojar mixture has significantly decreased solubility in organic solvents, compared to the 2− charged analogs in (Bu_4_N^+^)_2_[CO_3_⊂{Cu^II^(*μ*-OH)(*μ*-pz)}_n_]^2−^. Thus, the neutral nanojar mixture only dissolves well in solvents such as DMF, nitrobenzene and THF, but not in toluene, chlorobenzene, butyl acetate, methanol, acetone and dioxane, which are good solvents for the 2− charged nanojars. Finally, the peak at *m/z* = 2690 corresponding to [{Cu_2_O(phen)_3_CO_3_}CO_3_⊂{Cu(*μ*-OH)(*μ*-pz)}_31_]^2−^ clearly demonstrates the existence of expanded nanojars in solution.

**Figure 9:**
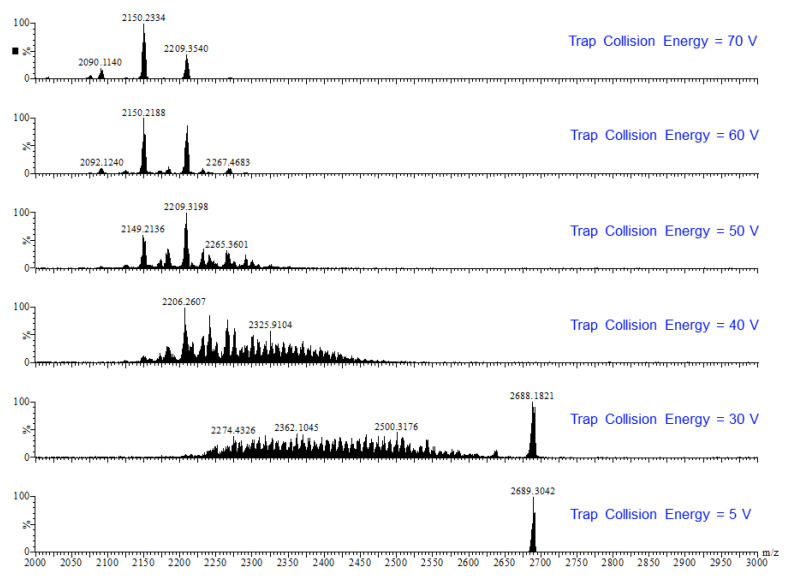
ESI-MS-MS(–) spectra of the isolated peak with *m/z* = 2690.

**Figure 10:**
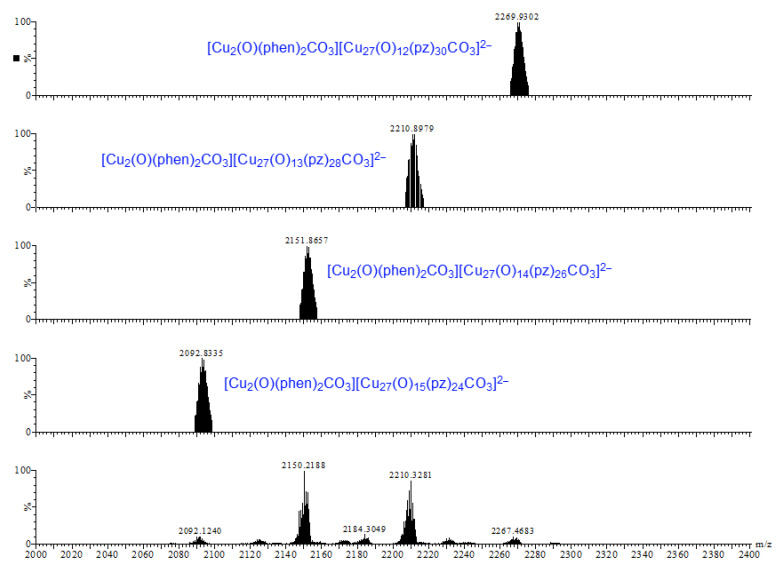
Comparison of the calculated (**upper four**) and observed (**bottom**) daughter-peaks of the isolated peak with *m/z* = 2690.

Earlier tandem mass spectrometry (MS-MS) studies showed that the various [CO_3_⊂{Cu(*μ*-OH)(*μ*-pz)}_n_]^2−^ nanojar species shrink as the trap collision energy is increased, by losing neutral Cu_5_(OH)_10_(Hpz)_10−y_(H_2_O)_(n+y−20)/2_ fragments (*y* = 4–12; y has the same parity as *n* [1]. Thus, four to five shrunken daughter-nanojar species of the formula [Cu_n−5_O_(n − y)/2_(pz)_n + y − 10_CO_3_]^2−^ were observed for each parent nanojar. Similarly, the peak at *m/z* = 2690 corresponding to [{Cu_2_O(phen)_3_CO_3_}CO_3_⊂{Cu(*μ*-OH)(*μ*-pz)}_31_]^2−^ gradually disappears upon increasing the trap collision energy, by losing a neutral Cu_4_(OH)_y_(Hpz)_8 − y_(H_2_O)_(31 − y)/2_(phen) fragment and giving rise to peaks at *m/z* 2092, 2151, 2269 and 2210 (Figure 9), which correspond to [Cu_27_O_(31 − y)/2_(pz)_31 + y − 8_CO_3_]^2−^ (*y* = 1, 3, 5, 7) species (Figure 10). So far, these shrunken daughter-nanojars have only been observed by mass spectrometry and have not been isolated; therefore, their detailed structure is yet unknown. As with other nanojars, a peak at *m/z* = 198, corresponding to [Cu^I^(pz)_2_]^−^, is also observed in the tandem mass spectrum.

**Figure 11:**
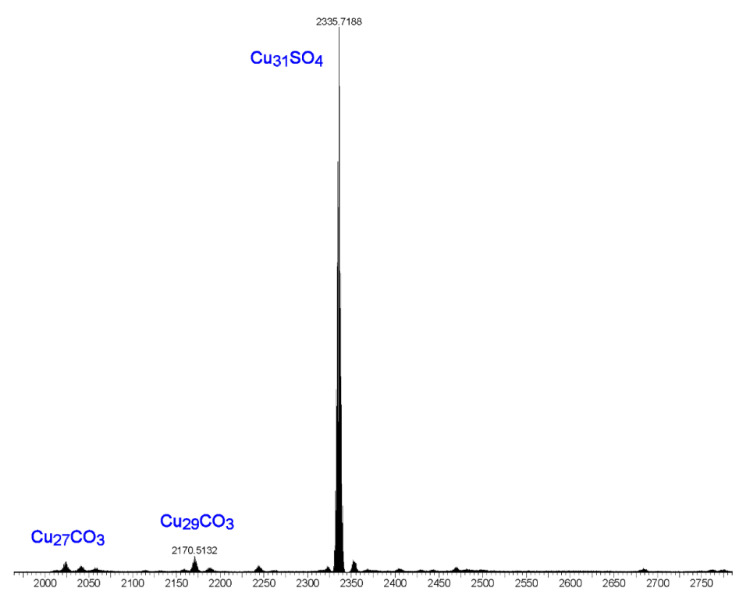
ESI-MS(–) spectrum in DMF of the product obtained from the reaction of copper sulfate, pyrazole, sodium hydroxide and 1,10-phenanthroline.

The following observations further support the assumption that the [Cu_2_(phen)_2_CO_3_]^2+^ moiety binds only to the Cu_9_-ring in nanojars. The ESI-MS(–) spectrum of the product obtained from the reaction of copper sulfate, pyrazole, sodium hydroxide and 1,10-phenanthroline is shown in Figure 11. The major peak in this spectrum corresponds to [SO_4_⊂{Cu(*μ*-OH)(*μ*-pz)}_31_]^2−^ (Cu_31_SO_4_), assumingly derived from [{Cu_2_(phen)_2_CO_3_}SO_4_⊂{Cu(*μ*-OH)(*μ*-pz)}_31_]. Smaller peaks are observed at *m/z* 2023 (Cu_27_CO_3_) and *m/z* 2171 (Cu_29_CO_3_). These latter species formed as side-products upon absorption of small amounts of atmospheric CO_2_ during the reaction and explain the serendipitous formation of a few crystals of **2**. Noteworthy is the absence of significant peaks at *m/z* 2041 (Cu_27_SO_4_), *m/z* 2115 (Cu_28_SO_4_), *m/z* 2189 (Cu_29_SO_4_) and *m/z* 2262 (Cu_30_SO_4_). As shown earlier, Cu_27_SO_4_ and Cu_29_SO_4_ species form in very small amounts under similar reaction conditions from copper sulfate, pyrazole, sodium hydroxide and tetrabutylammonium hydroxide in THF, whereas Cu_30_SO_4_ was not observed at all (the major species observed were Cu_28_SO_4_ and Cu_31_SO_4_) [1]. The absence of a peak at *m/z* 2115, corresponding to Cu_28_SO_4_, suggests that [{Cu_2_(phen)_2_CO_3_}SO_4_⊂{Cu(*μ*-OH)(*μ*-pz)}_28_] nanojars do not form, probably due to the fact that the Cu_28_SO_4_ nanojar (Cu_6_+Cu_12_+Cu_10_) lacks a Cu_9_-ring and cannot accommodate the Cu_2_(phen)_2_CO_3_ moiety.

## 3. Materials and Methods

All commercially available chemicals were used as received. Reactions were carried out in closed vessels, but not under strictly air-free conditions.

*Synthesis of [{Cu_2_(phen)_2_CO_3_}CO_3_⊂{Cu(μ-OH)(μ-pz)}_n_] (n = 27, 29, 30, 31)*. Cu(NO_3_)_2_·2.5H_2_O (0.4652 g, 2.00 mmol), pyrazole (0.132 g, 1.94 mmol), NaOH (0.155 g, 3.88 mmol), 1,10-phenanthroline (0.037 g, 0.21 mmol) and Na_2_CO_3_·H_2_O (0.2481, 2.00 mmol) were stirred for six days in 15 mL of THF. The reaction mixture was filtered, and the solvent was left to evaporate. The solid product obtained was washed with water, methanol and acetone and was dried under high vacuum to yield 0.283 g of a dark blue powder.

*Synthesis of [{Cu_2_(phen)_2_CO_3_}SO_4_⊂{Cu(μ-OH)(μ-pz)}_31_]*. This compound was prepared as described above, using CuSO_4_·5H_2_O (1.0000 g, 4.00 mmol), pyrazole (0.2640 g, 3.88 mmol), NaOH (0.3100 g, 7.75 mmol) and 1,10-phenanthroline (0.0740 g, 0.41 mmol) in 30 mL of THF. Yield: 0.5348 g dark blue powder.

### 3.1. Mass Spectrometry

Mass spectrometric analysis of the nanojars was performed with a Waters Synapt G1 HDMS instrument using electrospray ionization (ESI). 10^−4^–10^−5^ M solutions were prepared in *N*,*N*-dimethylformamide (DMF). Samples were infused by a syringe pump at 5 μL/min, and nitrogen was supplied as nebulizing gas at 500 L/h. The electrospray capillary voltage was set to –2.5 or +3.0 kV, respectively, with a desolvation temperature of 150 °C. The sampling and extraction cones were maintained at 40 V and 4.0 V, respectively, at 80 °C. The MS/MS conditions were the same, except the transfer collision energy was 5 V and the trap collision energies were 5, 30, 40, 50, 60 and 70 V.

### 3.2. X-ray Crystallography

A few single-crystals of **2** were grown from a nitrobenzene solution by heptane vapor diffusion. Once removed from the mother liquor, the crystals are very sensitive to solvent loss at ambient conditions and were mounted quickly under a cryostream (100 K) to prevent decomposition. X-ray diffraction data were collected at 100 K from a single-crystal mounted atop a glass fiber under Paratone-N oil with a Bruker SMART APEX II diffractometer using graphite-monochromated Mo-K*α* (*λ* = 0.71073 Å) radiation. The structure was solved by employing SHELXTL direct methods and refined by full-matrix least squares on *F*^2^ using the APEX2 v2014.9-0 software package [30]. C–H hydrogen atoms were placed in idealized positions and refined using the riding model. Hydroxyl and water H atom positions were located from difference density maps and were refined with O–H distance restraints of 0.82(2) Å. A pyrazolate ligand was refined as disordered. The two disordered moieties were restrained to have similar geometries. U_ij_ components of ADPs for disordered atoms closer to each other than 2.0 Å were restrained to be similar. Subject to these conditions, the occupancy ratio refined to 0.805(13)/0.195(4). The oxygen atoms of the carbonate ion were refined as disordered. The two disordered moieties were restrained to have similar geometries. U_ij_ components of ADPs for disordered atoms closer to each other than 2.0 Å were restrained to be similar. Subject to these conditions, the occupancy ratio refined to 0.913(4)/0.087(4). Three nitrobenzene solvate molecules were disordered with two alternative orientations (one by two-fold symmetry, two in general positions), one with three orientations, and one was disordered with a heptane molecule. The disordered nitrobenzene moieties were restrained to have similar geometries (SAME commands). C926 of one nitrobenzene moiety was restrained to be coplanar with its neighboring atoms. Bond distances within the heptane molecule were restrained to be similar to each other (SADI command). U_ij_ components of ADPs for disordered atoms closer to each other than 2.0 Å were restrained to be similar. Subject to these conditions, the occupancy rates refined to 0.910(4)/0.090(4) for the two moieties of the nitrobenzene of N60, to 0.489(14)/0.511(14) for the two moieties of the nitrobenzene of N62, to 0.502(3)/0.313(3)/0.185(3) for the three moieties of the nitrobenzene of N64 and to 0.765(6)/0.235(6) for the disorder of heptane and nitrobenzene (in favor of heptane). A thermal ellipsoid plot of the crystal structure is shown in Figure 7.

*Summary of the crystallographic data*. Chemical formula, C_152.76_H_171.91_Cu_29_N_64.74_O_47.47_; formula weight, 5517.12; crystal system, triclinic; space group, P ī (No. 2); *a* = 14.7855(3) Å; *b* = 20.6120(4) Å; *c* = 31.7417(6) Å; *α* = 95.026(1)°; *β* = 92.124(1)°; *γ* = 93.936(1); *V* = 9604.6(3) Å^3^; *Z* = 2; *D*_calc_ = 1.908 g/cm^3^; *μ* = 3.225 mm^−1^; no. of reflns collected, 299360; no. of unique reflns, 39404; no. of obsd reflns (*I* > 2*σ*(*I*)), 29554; R(int), 0.0723; data/parameters/restrains, 39404/3255/2672; goodness-of-fit (on *F*^2^): 1.032; R(*F*) (*I* > 2*σ*(*I*)), 0.0384; R_w_(*F*) (*I* > 2*σ*(*I*)), 0.0793; R(*F*) (all data), 0.0641; R_w_(*F*) (all data), 0.0891; residual electron density, max/min (*e*/Å^3^), 2.122/–1.143. Crystallographic data for **2**(C_6_H_5_NO_2_)_6.74_(C_7_H_16_)_0.76_ were deposited with the Cambridge Crystallographic Data Center (CCDC 2078120). Copies of the data can be obtained free of charge at http://www.ccdc.cam.ac.uk/products/csd/request (accesed on 20 May 2021).

## 4. Conclusions

In summary, we present a new, expanded nanojar of the formula [CO_3_⊂{Cu_29_(*μ*-OH)_27_(*μ*-pz)_27_(phen)_2_(*μ*_4_-CO_3_)(H_2_O)}], which is able to bind two carbonate ions, compared to only one carbonate ion in previously reported nanojars [CO_3_⊂{Cu(*μ*-OH)(*μ*-pz)}_n_]^2−^ (*n* = 27, 29, 30, 31). Single-crystal X-ray crystallographic studies in the solid state show that the new nanojar is an extension of the [CO_3_⊂{Cu(*μ*-OH)(*μ*-pz)}_6+12+9_]^2−^ motif, with a [Cu_2_(phen)_2_CO_3_]^2+^ moiety bound to the Cu_9_-ring of the nanojar. Upon binding of this additional moiety, one OH^−^ group of the Cu_9_-ring is displaced by an O-atom of the second carbonate ion and becomes a bridging ligand for the Cu_2_-moiety. Thus, the expanded nanojar can also be described as [{Cu_2_(*μ*-OH)(phen)_2_(*μ*_4_-CO_3_)}CO_3_⊂{Cu_27_(*μ*-OH)_26_(*μ*-pz)_27_}]. Solution studies by electrospray ionization mass spectrometry indicate that homologous nanojar species based on [CO_3_⊂{Cu(*μ*-OH)(*μ*-pz)}_n_]^2−^ (*n* = 29, 30, 31) also form in the reaction of copper nitrate, pyrazole, sodium hydroxide, sodium carbonate and 1,10-phenanthroline. Although the expanded nanojars described above cannot be observed directly by ESI-MS due to their overall neutral charge, ionization by loss of the [Cu_2_(phen)_2_CO_3_]^2+^ moiety does lead to [CO_3_⊂{Cu(*μ*-OH)(*μ*-pz)}_n_]^2−^ (*n* = 27, 29, 30, 31) daughter-species. Additionally, a peak in the mass spectrum corresponding to [{Cu_2_O(phen)_3_CO_3_}CO_3_⊂{Cu(*μ*-OH)(*μ*-pz)}_31_]^2−^ does provide direct evidence and demonstrates the existence of expanded nanojars in solution.

## Figures and Tables

**Table 1:** Selected bond lengths for **2**.

Cu1–O1 1.906(3)	Cu8–N14 1.964(4)	Cu16–O16 1.928(3)	Cu23–N45 1.946(3)
Cu1–O6 1.957(3)	Cu8–N815 2.06(2)	Cu16–N31 1.964(3)	Cu23–O23 1.951(3)
Cu1–N12 1.983(4)	Cu9–O9 1.910(3)	Cu16–N30 1.975(4)	Cu23–N44 1.963(3)
Cu1–N1 2.025(3)	Cu9–O8 1.935(3)	Cu17–O17 1.908(3)	Cu24–O24 1.891(3)
Cu2–O1 1.928(3)	Cu9–N16 1.961(6)	Cu17–O16 1.934(3)	Cu24–N46 1.948(3)
Cu2–O2 1.939(3)	Cu9–N17 1.962(3)	Cu17–N32 1.954(4)	Cu24–O23 1.950(3)
Cu2–N3 1.982(3)	Cu9–N816 2.00(2)	Cu17–N33 1.974(4)	Cu24–N47 1.968(4)
Cu2–N2 2.000(3)	Cu10–O10 1.909(3)	Cu18–O17 1.925(3)	Cu25–O24 1.930(3)
Cu2–O11 2.411(3)	Cu10–O9 1.939(3)	Cu18–O18 1.934(3)	Cu25–O25 1.937(3)
Cu3–O2 1.928(3)	Cu10–N18 1.961(3)	Cu18–N34 1.963(3)	Cu25–N49 2.001(3)
Cu3–O3 1.940(3)	Cu10–N19 1.983(3)	Cu18–N35 1.976(4)	Cu25–N48 2.005(4)
Cu3–N4 1.998(3)	Cu11–O11 1.921(3)	Cu19–O27 1.934(3)	Cu25–O14 2.326(3)
Cu3–N5 2.003(3)	Cu11–O10 1.931(3)	Cu19–O19 1.958(3)	Cu26–O25 1.893(3)
Cu4–O3 1.938(3)	Cu11–N20 1.965(3)	Cu19–N37 1.992(4)	Cu26–N51 1.946(4)
Cu4–O4 1.941(3)	Cu11–N21 1.967(3)	Cu19–N54 2.000(3)	Cu26–O26 1.950(3)
Cu4–N7 1.981(3)	Cu12–O11 1.924(3)	Cu19–O18 2.399(3)	Cu26–N50 1.975(4)
Cu4–N6 2.010(4)	Cu12–O12 1.932(3)	Cu20–O19 1.919(3)	Cu27–O27 1.905(3)
Cu5–O5 1.934(3)	Cu12–N23 1.956(4)	Cu20–N38 1.970(4)	Cu27–O26 1.938(3)
Cu5–O4 1.941(3)	Cu12–N22 1.966(3)	Cu20–N39 1.989(4)	Cu27–N53 1.961(4)
Cu5–N8 1.996(3)	Cu13–O13 1.914(3)	Cu20–O20 1.998(3)	Cu27–N52 1.964(3)
Cu5–N9 2.002(4)	Cu13–O12 1.933(3)	Cu20–O40 2.419(3)	Cu28–O28 1.922(3)
Cu6–O5 1.916(3)	Cu13–N24 1.953(3)	Cu21–O21 1.911(3)	Cu28–O30 1.954(3)
Cu6–O6 1.956(3)	Cu13–N25 1.975(4)	Cu21–N40 1.958(4)	Cu28–N56 2.022(4)
Cu6–N11 1.991(3)	Cu14–O14 1.914(3)	Cu21–N41 1.973(4)	Cu28–N55 2.023(4)
Cu6–N10 2.006(3)	Cu14–O13 1.926(3)	Cu21–O20 2.007(3)	Cu28–O26 2.296(3)
Cu7–O18 1.905(3)	Cu14–N26 1.959(4)	Cu21–O40 2.432(3)	Cu29–O28 1.919(3)
Cu7–O7 1.921(3)	Cu14–N27 1.960(3)	Cu22–O22 1.927(3)	Cu29–O29 1.942(3)
Cu7–N13 1.954(4)	Cu15–O14 1.923(3)	Cu22–N42 1.969(4)	Cu29–N57 2.019(4)
Cu7–N36 1.963(3)	Cu15–O15 1.925(3)	Cu22–O21 1.975(3)	Cu29–N58 2.026(3)
Cu8–O7 1.907(3)	Cu15–N29 1.966(3)	Cu22–N43 2.004(4)	Cu29–O23 2.269(3)
Cu8–O8 1.941(3)	Cu15–N28 1.969(4)	Cu22–O10 2.318(3)	
Cu8–N15 1.943(6)	Cu16–O15 1.916(3)	Cu23–O22 1.894(3)	

**Table 2:** Hydrogen bonding data for **2**. O1–O6: Cu_6_-ring; O7–O18: Cu_12_-ring; O19–O27: Cu_9_-ring; O28: Cu_2_(OH)(phen)_2_ unit; O31–O33 and O931–O933: central carbonate (disordered, 91/9).

D–H···A	D–H (Å)	H···A (Å)	D···A (Å)	D–H–A (°)
O1–H1O···O33	0.80(2)	1.89(2)	2.687(5)	170(5)
O2–H2O···O33	0.78(2)	2.15(2)	2.915(5)	165(5)
O2–H2O···O933	0.78(2)	1.91(2)	2.657(5)	158(5)
O3–H3O···O31	0.80(2)	2.34(2)	3.124(5)	166(5)
O4–H4O···O32	0.80(2)	2.28(3)	3.036(5)	158(5)
O4–H4O···O931	0.80(2)	1.92(3)	2.678(5)	157(5)
O5–H5O···O32	0.80(2)	1.88(2)	2.677(5)	175(5)
O6–H6O···O932	0.80(2)	1.93(3)	2.713(5)	166(5)
O7–H7O···O19	0.81(2)	2.11(2)	2.914(5)	172(5)
O8–H8O···O6	0.80(2)	1.92(2)	2.716(4)	178(6)
O9–H9O···O21	0.81(2)	2.01(2)	2.818(4)	172(5)
O10–H10O···O1	0.81(2)	1.93(2)	2.731(4)	171(5)
O11–H11O···O22	0.81(2)	1.92(2)	2.731(4)	175(5)
O12–H12O···O2	0.80(2)	2.00(2)	2.787(4)	172(5)
O13–H13O···O24	0.79(2)	1.95(2)	2.743(5)	177(6)
O14–H14O···O3	0.80(2)	1.94(2)	2.721(4)	168(5)
O15–H15O···O25	0.81(2)	1.91(2)	2.722(4)	178(5)
O16–H16O···O4	0.80(2)	1.96(2)	2.764(4)	174(5)
O17–H17O···O27	0.81(2)	1.97(2)	2.767(4)	171(5)
O18–H18O···O5	0.80(2)	1.94(2)	2.736(4)	172(5)
O19–H19O···O32	0.81(2)	2.17(2)	2.949(5)	161(5)
O19–H19O···O932	0.81(2)	2.27(3)	3.055(5)	166(5)
O21–H21O···O33	0.80(2)	2.22(3)	2.973(5)	156(5)
O21–H21O···O932	0.80(2)	2.30(3)	3.088(5)	167(5)
O22–H22O···O33	0.80(2)	1.90(2)	2.696(4)	170(5)
O22–H22O···O933	0.80(2)	2.18(2)	2.907(4)	151(5)
O23–H23O···O31	0.79(2)	2.47(3)	3.195(5)	153(5)
O23–H23O···O933	0.79(2)	1.95(3)	2.730(5)	167(5)
O24–H24O···O31	0.80(2)	1.95(2)	2.737(5)	168(5)
O24–H24O···O933	0.80(2)	2.29(2)	3.023(5)	153(5)
O25–H25O···O31	0.80(2)	1.90(3)	2.682(5)	164(5)
O25–H25O···O931	0.80(2)	2.24(3)	2.985(5)	156(5)
O26–H26O···O31	0.80(2)	2.33(3)	3.045(5)	150(5)
O26–H26O···O931	0.80(2)	1.90(3)	2.693(5)	177(5)
O27–H27O···O32	0.80(2)	1.96(3)	2.741(5)	163(5)
O27–H27O···O931	0.80(2)	2.14(3)	2.893(5)	155(5)
O28–H28O···O31	0.82(2)	2.11(3)	2.895(5)	159(5)
O40–H40O···O8	0.82(2)	1.87(2)	2.684(5)	174(6)

## Data Availability

Not applicable.

## Sample Availability

Samples of the compounds are not available from the authors.
